# Echocardiography and inflammatory biomarkers for predicting mortality and major adverse cardiovascular events in type 1 diabetes

**DOI:** 10.1186/s12933-025-03071-2

**Published:** 2026-02-03

**Authors:** Hashmat Sayed Zohori Bahrami, Peter Godsk Jørgensen, Jens Dahlgaard Hove, Ulrik Dixen, Line Jee Hartmann Rasmussen, Jesper Eugen-Olsen, Peter Rossing, Magnus T. Jensen

**Affiliations:** 1 Department of Clinical and Translational Research, Steno Diabetes Center Copenhagen, Borgmester Ib Juuls Vej 83, 2730 Herlev, Denmark; 2 Department of Cardiology, Copenhagen University Hospital, Amager and Hvidovre, Kettegård Alle 30, 2650 Hvidovre, Denmark; 3 Department of Cardiology, Copenhagen University Hospital, Herlev and Gentofte, Borgmester Ib Juuls Vej 1, 2730 Herlev, Denmark; 4 Department of Clinical Research, Copenhagen University Hospital, Amager and Hvidovre, Kettegård Alle 36, 2650 Hvidovre, Denmark; 5 Department of Clinical Medicine, Faculty of Health and Medical Sciences, University of Copenhagen, Blegdamsvej 3B, 2200 Copenhagen, Denmark; 6 Department of Psychology and Neuroscience, Duke University, 2020 W Main St, Durham, NC 27708 USA; 7 NIHR Barts Biomedical Centre, William Harvey Research Institute, Queen Mary University London, Charterhouse Square, London, EC1M 6BQ UK

**Keywords:** Echocardiography, Diabetes, Inflammation, Biomarkers, RiskAssessment

## Abstract

**Background/aims:**

Current clinical risk tools in type 1 diabetes do not include left ventricular dysfunction or inflammation, potentially limiting early risk detection. We aimed to evaluate the associations and predictive value of combining echocardiography with inflammatory biomarkers for mortality and major adverse cardiovascular events (MACE).

**Methods:**

In a prospective cohort of individuals with type 1 diabetes without known cardiovascular disease, we evaluated whether subclinical left ventricular dysfunction, defined by an elevated ratio of early mitral inflow velocity to early diastolic mitral annular velocity (E/e′) or impaired global longitudinal strain (GLS), combined with elevated levels of an inflammatory biomarker (interleukin-6 [IL-6], soluble urokinase-plasminogen-activator-receptor [suPAR], or high-sensitivity C-reactive-protein [hsCRP]), was associated with all-cause mortality and MACE. Cox models were adjusted for all 10 variables included in the Steno T1 Risk Engine variables: age, sex, systolic blood pressure, duration of diabetes, HbA1c, low-density lipoprotein, estimated glomerular filtration rate, albuminuria status, smoking, and physical activity. C-statistics and net reclassification improvement were assessed.

**Results:**

Among 876 participants (51% male, median age 50 years), 114 deaths occurred over 14.5 years of follow-up. Elevated E/e’ combined with IL-6 or suPAR, but not hsCRP, was independently associated with mortality. Compared with individuals with E/e’ <8 and non-elevated IL-6, the hazard ratio (HR) for E/e’ 8–13 with elevated IL-6 was 2.5 (95% CI 1.4 to 4.6, *P* < 0.01), and for E/e′ ≥13 with elevated IL-6 was 3.4 (1.5–7.6; *P* < 0.01). Corresponding HRs for suPAR were 2.4 (1.2 to 4.7, *P* < 0.01) and 3.9 (1.8 to 8.5, *P* < 0.01). Adding E/e′ and an inflammatory biomarker increased the C-statistic from 0.839 (Steno T1 Risk Engine alone) to 0.887 (E/e’ and IL6) and 0.868 (E/e’ and suPAR). Findings were similar for GLS and with MACE as the outcome.

**Conclusions:**

Echocardiography combined with inflammatory biomarkers synergistically identifies individuals with type 1 diabetes, without known cardiovascular disease, who are at high risk of mortality and MACE.

**Supplementary Information:**

The online version contains supplementary material available at 10.1186/s12933-025-03071-2.

## Introduction

Despite major advances in diabetes care, life expectancy in individuals with type 1 diabetes remains ~ 13 years shorter than in the general population, largely driven by excess cardiovascular mortality [1, 2]. This gap highlights the urgent need for improved risk stratification strategies to identify individuals at high risk of premature death [3]. Although existing clinical risk scores capture many traditional predictors, they lack two central pathophysiological features of diabetes-related cardiovascular disease: myocardial dysfunction and chronic inflammation [4].

Heart failure is increasingly recognized as one of the most prevalent and morbid cardiovascular complications of diabetes [5–7]. Subclinical left ventricular dysfunction, which often precedes overt heart failure, can be detected using sensitive echocardiographic markers. Among these, E/e’ (the ratio of early diastolic transmitral inflow velocity to early diastolic mitral annular tissue velocity) is a widely accepted, non-invasive surrogate of left ventricular filling pressure and is frequently elevated in diabetic cardiomyopathy [8–10]. Global longitudinal strain (GLS) represents another established measure of subclinical left ventricular dysfunction [11]. We have previously shown that both diastolic and systolic impairments are underrecognized and are associated with increased risk of all-cause mortality and major adverse cardiovascular events (MACE) in individuals with type 1 diabetes without known heart disease [12, 13].

Chronic low-grade inflammation represents another hallmark of T1D and constitutes another shared pathophysiological mechanism with atherosclerosis and heart failure, two major contributors to diabetes-related mortality [14–16]. Established biomarkers of systemic inflammation include high-sensitivity C-reactive protein (hsCRP), interleukin-6 (IL-6), and soluble urokinase plasminogen activator receptor (suPAR). These biomarkers have demonstrated robust prognostic value for cardiovascular disease and mortality in both cardiovascular and general populations [17–19]. We recently showed that IL-6 and suPAR independently predict all-cause mortality and MACE in individuals with type 1 diabetes [20].

However, the prognostic value of combining echocardiographic assessment with an inflammatory biomarker has not previously been investigated. Therefore, we aimed to investigate the long-term (~ 15 years) prognostic value of E/e’ and GLS, both alone and in combination with three biomarkers of inflammation (IL-6, suPAR, and hsCRP), in individuals with type 1 diabetes without known cardiovascular disease and with left ventricular ejection fraction (LVEF) > 50%.

We hypothesized that: (A) Elevated E/e’, impaired GLS, and elevated inflammatory biomarker levels are associated with an increased risk of all-cause mortality and MACE, independent of each other and conventional risk factors; (B) risk is further augmented when these echocardiographic abnormalities coexist with elevated inflammatory biomarker levels; and (C) this combined approach improves discrimination beyond an established clinical risk model.

## Methods

### Study population

The Thousand & 1 study is a prospective clinical cohort study comprising 1,093 individuals with type 1 diabetes and no known heart disease [12]. Participants were recruited from the Steno Diabetes Center Copenhagen and examined between April 2010 and April 2012. Eligible individuals were ≥ 18 years of age, attended the outpatient clinic, were willing to participate, and had no history of heart disease. Heart disease was defined as atrial fibrillation or flutter; moderate valvular disease (including mitral annular calcification and mitral regurgitation) identified during echocardiographic examination; heart failure; coronary artery disease (including prior myocardial infarction, stable angina, percutaneous coronary intervention, or coronary artery bypass grafting); left bundle branch block; congenital heart disease; pacemaker implantation; or implantable cardioverter-defibrillator implantation. All such conditions constituted exclusion criteria. Further details of the study design have been reported previously [12]. For the present study, 52 individuals were excluded due to a prior diagnosis of stroke, 26 due to the use of anti-inflammatory or immunomodulatory medication at baseline, 62 due to LVEF ≤ 50%, three due to missing E/e′ measurements, and 74 due to missing suPAR and/or IL-6 measurements.

The study was conducted in accordance with the second Helsinki Declaration and approved by the regional ethics committee (H-3-2009-139 and H-21058624) and the Danish Data Protection Agency (00934-Geh-2010-003 and P-2021-719). All participants provided written informed consent [12].

### Inflammatory biomarker measurements

Biochemical data, including HbA1c, creatinine, and albuminuria status, were retrieved from electronic patient records corresponding to the outpatient visit closest to study inclusion (maximum ± 4 months) [12]. Serum samples for the measurement of suPAR, IL-6, and hsCRP were collected at baseline between 2010 and 2012 and stored at −80 °C until analysis in 2022 [20, 21]. Samples underwent up to two freeze-thaw cycles. All three biomarkers are known to be stable during long-term frozen storage.

*Serum IL-6* concentrations (pg/mL) were measured using the Human IL-6 Quantikine ELISA Kit (R&D Systems, Minneapolis, MN, USA) according to the manufacturer’s instructions. The assay’s detection limit was 1.0 pg/mL, and the upper limit of quantification was 300 pg/mL. Manufacturer-reported coefficients of variation were 1.6–4.2% for intra-assay and 3.3–6.4% for inter-assay variability.

*Serum suPAR* concentrations (ng/mL) were analyzed using the suPARnostic^®^ AUTO Flex ELISA (ViroGates A/S, Birkerød, Denmark) according to the manufacturer’s instructions. The assay’s detection limit was 0.4 ng/mL, and the upper limit of quantification was 15 ng/mL. Manufacturer-reported coefficients of variation were 2.8% for intra-assay and 9.2% for inter-assay.

*Serum hsCRP* concentrations (mg/L) were measured using a Cobas 6000 analyzer with c501 modules (Roche Diagnostics, GmbH, D-68298 Mannheim, Germany) according to the manufacturer’s instructions. The assay’s detection limit was 0.15 mg/L. Manufacturer-reported coefficients of variation were 0.4–1.6% for intra-assay and 1.3–8.4% for inter-assay.

### Clinical data and echocardiography

All participants completed a standardized questionnaire detailing smoking status, physical activity, and medication use. Sex was defined as binary biological sex assigned at birth (male or female), as recorded in medical records. Resting blood pressure was measured in the supine position. Echocardiography was then performed using a Vivid-7 ultrasound scanner (General Electric Vingmed Ultrasound AS, Horten, Norway) and analyzed offline using EchoPAC software (version BT11, General Electric) [12]. Three consecutive cardiac cycles were recorded. Mitral peak early diastolic (E) inflow velocity was measured in the four-chamber view using pulsed-wave-Doppler, with the sample volume placed between the tips of the mitral valve leaflets [22]. Early diastolic mitral annular tissue velocity (e′) was obtained using pulsed-wave tissue Doppler imaging at the lateral mitral annulus in the apical four-chamber view [22]. From these, E/e’ was calculated. Left ventricular GLS was measured using two-dimensional speckle-tracking echocardiography and calculated as the average of measurements from the three apical views [13]. LVEF was determined using the biplane Simpson’s method [23].

### Endpoints and follow-up

Follow-up was complete for all participants (100%) and concluded on 17th October 2024. Vital status was ascertained through linkage with the Danish National Registries. The primary outcome was all-cause mortality. The secondary outcome was MACE, defined as a composite of all-cause mortality or incident hospitalization for ischemic heart disease (ICD-10: DI200, DI21, DI23-24), heart failure (ICD-10: DI110, DI130, DI132, DI50, DI42), or stroke (ICD-10: DI60-66, DI693-694, DG45). Investigators performing echocardiographic analyses and biomarker measurements were blinded to outcome data, as all assessments were completed prior to outcome ascertainment.

### Statistical analysis

Continuous variables are presented as medians with interquartile ranges (IQRs) and were compared using the Wilcoxon rank-sum test. Categorical variables are presented as counts and were percentages and compared using the chi-squared test.

To examine the association between echocardiographic parameters, inflammatory biomarkers, and the outcomes, E/e′ was categorized, and GLS and the inflammatory biomarkers were dichotomized. E/e′ was stratified into three clinically meaningful categories (< 8, 8–13, and ≥ 13) based on clinical guidelines [8, 24]. GLS was dichotomized at ≥ 16% based on guideline recomendations [11]. hsCRP was dichotomized at ≥ 2.0 mg/L, consistent with cut-offs used in landmark clinical trials evaluating cardiovascular risk [16]. For IL-6 and suPAR, no such trial-based thresholds exist. Therefore, we performed receiver operating characteristic curve analyses using 12-year all-cause mortality as the outcome to determine optimal cut-offs. Thresholds for IL-6 (≥ 1.9 pg/mL) and suPAR (≥ 3.2 ng/mL) were identified by maximizing Youden’s index and applied for subsequent dichotomization.

Cumulative survival incidence curves were constructed, and differences between groups were compared using the log-rank test. Cox proportional hazards regression models were used to estimate hazard ratios (HRs) for E/e′, both individually and in combination with elevated inflammatory biomarkers, in association with all-cause mortality and MACE. Multivariable models were adjusted for the 10 variables included in the Steno Type 1 Risk Engine: age, sex, systolic blood pressure, duration of diabetes, HbA1c, low-density lipoprotein, estimated glomerular filtration rate, albuminuria status, smoking status (current or former), and physical activity [4].

To evaluate the incremental prognostic value of echocardiographic parameters and inflammatory biomarkers, C-statistics were calculated for the Steno T1 Risk Engine (applied as published, without recalibration) alone and with the sequential addition of: (1) elevated E/e′ alone; (2) impaired GLS alone; (3) elevated suPAR alone; (4) elevated IL-6 alone; (5) elevated E/e′ combined with elevated suPAR or elevated IL-6; and (6) impaired GLS combined with elevated suPAR or elevated IL-6. Similarly, we calculated the continuous net reclassification improvement (NRI).

In sensitivity analyses, we dichotomized IL-6 and suPAR based on commonly applied thresholds for distinguishing healthy from non-healthy individuals: IL-6 at 5.2 pg/mL [25] and suPAR at 4.0 ng/mL [26]. Additional adjustments were made for statin use, angiotensin-converting enzyme inhibitor use, and angiotensin II receptor blocker use. Restricted cubic spline analyses were conducted to explore the continuous shape of associations, with adjustment for the multivariable model. We tested for interactions between sex and E/e′, GLS, IL-6, suPAR, and hsCRP.

No formal power calculation was performed, as the study used a well-characterized cohort previously used in multiple prognostic studies, with adequate follow-up duration and events to support robust analyses [13, 20, 27]. All hsCRP and suPAR values were above the assay detection limit. For IL-6, 67% of values were below the detection limit and were imputed at the assay’s lower limit of 1.0 pg/mL. All model assumptions were validated, including the proportionality of hazards. We report two-tailed P-values and 95% confidence intervals, considering *P* < 0.05 as statistically significant. R software version 4.1.0 (R Project for Statistical Computing, University of Economics and Business Administration, Wien, Austria) was used for all analyses.

## Results

### Baseline characteristics

Among the 876 individuals included in the analysis, 51% were male, and the median age was 50 years (IQR 39–60). Follow-up was complete (100%) for all participants. During a maximum follow-up of 14.5 years (IQR 12.7–14.0), 114 individuals (13%) died, and 172 (20%) experienced a MACE.

Median (IQR) were 7 (5–8) for E/e′, 18% (17–20) for GLS, 1.0 pg/mL (1.0-2.1) for IL-6, 2.8 ng/mL (2.3–3.5) for suPAR, and 1.7 mg/L (0.7–3.6) for hsCRP (Table 1). An E/e’ of 8–13 was observed in 227 individuals (26%) and an E/e’ ≥13 in 41 individuals (5%). Impaired GLS < 16% was present in 127 individuals (14%). Simultaneous elevation of E/e′ (≥ 8) and inflammatory biomarkers was observed in 95 individuals (11%) for IL-6 (≥ 1.9 pg/mL) and 142 individuals (16%) for suPAR (≥ 3.2 ng/mL). When combined with impaired GLS (< 16%), the corresponding numbers were 48 individuals (5%) for IL-6 and 54 individuals (6%) for suPAR.

Compared with individuals with both low E/e′ and non-elevated inflammatory biomarker levels, those with concurrent elevations of E/e′ and an inflammatory biomarker were older, more frequently female, had longer diabetes duration, higher systolic blood pressure, reduced kidney function, a more prevalent smoking history, and greater use of medications, irrespective of the specific biomarker evaluated (Table 1, Supplemental Table 1).

**Table 1:** Baseline characteristics by E/e′ combined with IL-6 or SuPAR

		(*n* = 876) All	(*n* = 471) E/e’ <8 & IL-6 < 1.9	(*n* = 95) E/e’ ≥8 & IL-6 ≥ 1.9	*P* value	(*n* = 446) E/e’ <8 & suPAR < 3.2	(*n* = 142) E/e’ ≥8 & suPAR ≥ 3.2	*P* value
Age, years, median (IQR)		50 [39–60]	44 [35–55]	60 [52–67]	< 0.001	43 [32–52]	61 [53–67]	< 0.001
Male sex, n (%)		447 (51)	259 (55)	43 (45)	0.039	255 (57)	50 (35)	< 0.001
Body mass index, kg/m^2^, median (IQR)		25 [23–28]	24 [23–27]	26 [24–29]	< 0.001	25 [23–28]	25 [23–28]	0.093
Diabetes duration, years, median (IQR)		25 [15–36]	20 [12–32]	37 [28–46]	< 0.001	20 [12–30]	38 [30–47]	< 0.001
Systolic blood pressure, mmHg, median (IQR)		132 [122–142]	128 [120–140]	138 [130–152]	< 0.001	128 [120–138]	142 [130–152]	< 0.001
eGFR, mL/min/1.73m^2^, median (IQR)		89 [ 76–102]	91 [ 80–104]	77 [59–91]	< 0.001	93 [ 82–107]	74 [57–90]	< 0.001
Hemoglobin A1c, mmol/L, median (IQR)		64 [57–74]	64 [56–74]	67 [58–74]	0.222	64 [56–74]	66 [60–75]	0.039
Hemoglobin A1c, %, median (IQR)		8.0 [7.4–8.9]	8.0 [7.3–8.9]	8.3 [7.4–8.9]	0.222	8.0 [7.3–8.9]	8.2 [7.6-9.0]	0.039
Total cholesterol, mmol/L, median (IQR)		4.8 [4.2–5.3]	4.7 [4.2–5.2]	4.8 [4.3–5.2]	0.401	4.7 [4.2–5.2]	4.8 [4.2–5.3]	0.558
Low-density lipoprotein, mmol/L, median (IQR)		2.5 [2.1-3.0]	2.6 [2.1-3.0]	2.4 [2.0-2.8]	0.047	2.6 [2.1–3.1]	2.4 [2.0-2.9]	0.024
Interleukin-6, pg/mL, median (IQR)		1.0 [1.0-2.1]	1.0 [1.0–1.0]	5.5 [3.1–9.1]	< 0.001	1.0 [1.0-1.4]	1.0 [1.0-5.2]	< 0.001
suPAR, ng/mL, median (IQR)		2.8 [2.3–3.5]	2.6 [2.2–3.3]	3.5 [2.8–4.9]	< 0.001	2.5 [2.1–2.8]	4.2 [3.6-5.0]	< 0.001
High-sensitivity CRP, mg/L, median (IQR)		1.7 [0.7–3.6]	1.2 [0.5–2.7]	3.2 [1.8–7.7]	< 0.001	1.3 [0.6–3.2]	2.3 [1.1–4.9]	< 0.001
Current smoking, n (%)		485 (55)	238 (51)	63 (66)	0.004	207 (46)	97 (68)	< 0.001
*Albuminuria, n (%)*					< 0.001			< 0.001
	Mildly increased	631 (72)	378 (80)	40 (42)		372 (83)	60 (42)	
	Moderately increased	76 (9)	22 (5)	24 (25)		10 (2)	37 (26)	
	Severely increased	169 (19)	71 (15)	31 (33)		64 (14)	45 (32)	
*Medications at inclusion, n (%)*								
	Statins	365 (42)	146 (31)	67 (71)	< 0.001	126 (28)	99 (70)	< 0.001
	ACE-I/ARB	387 (44)	156 (33)	71 (75)	< 0.001	128 (29)	105 (74)	< 0.001
	Beta-blockers	34 (4)	5 (1)	14 (15)	< 0.001	2 (0.1)	21 (15)	< 0.001
	Diuretics	217 (25)	70 (15)	54 (57)	< 0.001	49 (11)	80 (56)	< 0.001
*Echocardiography*								
	LVEF, %, median (IQR)	58 [55–61]	58 [55–61]	58 [54–62]	0.462	57 [55–61]	58 [55–62]	0.034
	GLS, %, median (IQR)	18 [17, 20]	19 [17, 20]	17 [16, 19]	< 0.001	19 [17, 20]	18 [16, 19]	< 0.001
	E/e’, median (IQR)	7 [5–8]	6 [5–7]	11 [ 9–12]	< 0.001	6 [5–7]	10 [ 9–12]	< 0.001
	e’ lateral, cm/s, median (IQR)	13 [10–16]	14 [12–17]	9 [ 8–10]	< 0.001	15 [12–17]	9 [ 7–10]	< 0.001
	Left atrial volume, mL/m²	29 [26–34]	29 [25–34]	31 [27–36]	0.025	29 [25–33]	30 [26–34]	0.177

*P* value for comparison between low-low and high-high groups

ACE-I, angiotensin-converting enzyme inhibitor ARB, angiotensin receptor blocker; CRP, C-reactive protein; eGFR, estimated glomerular filtration rate; GLS, global longitudinal strain; IL-6, interleukin-6; IQR, interquartile range; LVEF, left ventricular ejection fraction; suPAR, soluble urokinase plasminogen activator receptor

### Prognostic value of E/e’ and inflammatory biomarkers

In unadjusted analysis, the cumulative incidence of all-cause mortality increased across E/e′ strata, from 9% among individuals with E/e′ <8, to 25% for E/e′ 8–13, and 61% for E/e′ ≥13 (Supplemental Fig. 1a). Supplemental Fig. 1b-d shows that E/e’ stratified mortality risk even among individuals with elevated levels of IL-6, suPAR, or hsCRP. Similar patterns were observed when MACE was used as the outcome (Supplemental Fig. 1e-g). This indicates that E/e′ provides prognostic information that is at least partially independent of systemic inflammatory burden.

**Fig. 1:**
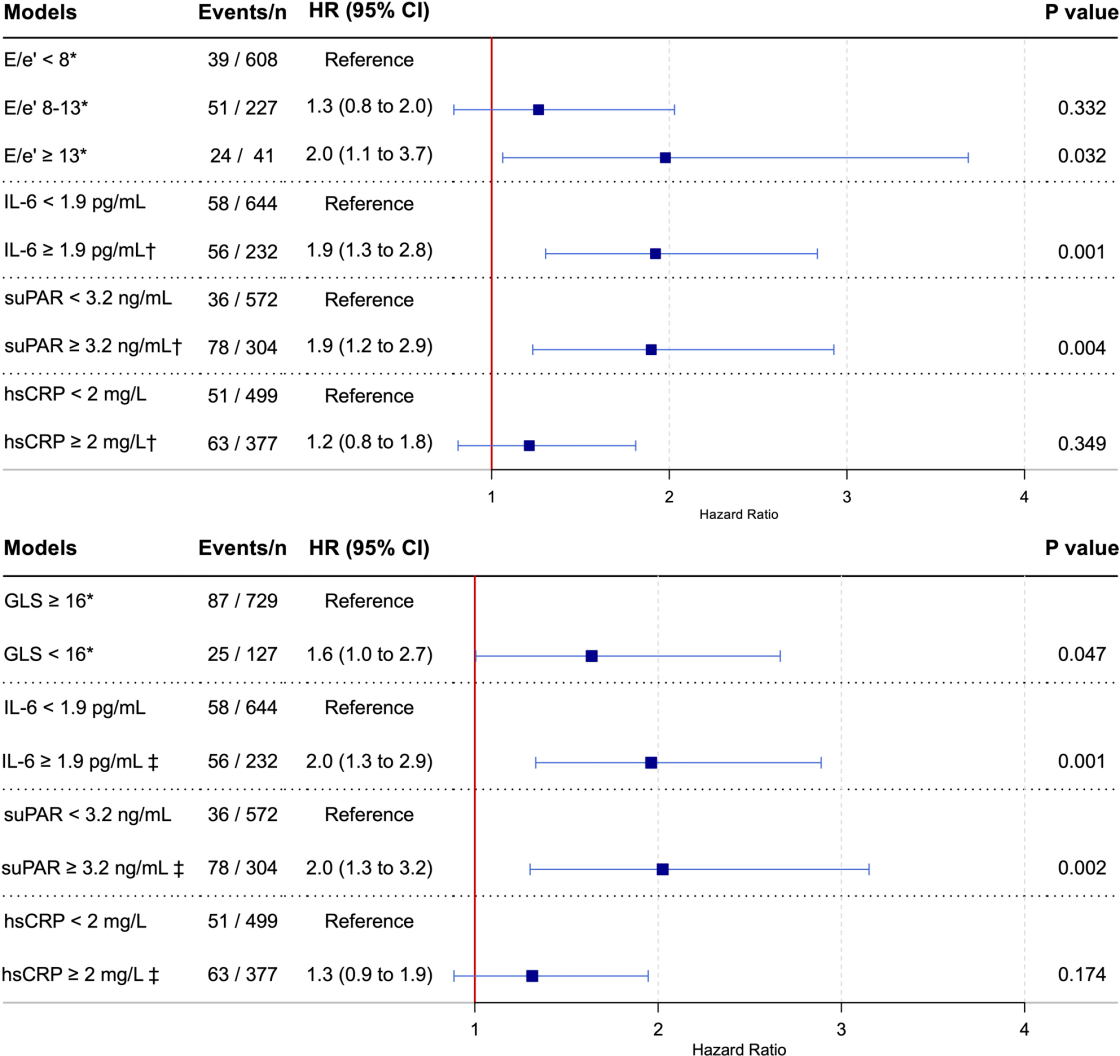
Independent associations of echocardiographic parameters and inflammatory biomarkers with all-cause mortality. *: Adjusted for IL-6, suPAR, hsCRP, and Steno T1 Risk Engine variables: age, sex, systolic blood pressure, duration of diabetes, HbA1c, low-density lipoprotein, estimated glomerular filtration rate, albuminuria status, smoking, and physical activity. †: Adjusted for E/e’ and Steno T1 Risk Engine variables. ‡: Adjusted for GLS and Steno T1 Risk Engine variables. CI = confidence interval; E/e’ = ratio of early mitral inflow velocity to early diastolic mitral annular velocity; GLS = global longitudinal strain; HR = hazard ratio; hsCRP = high-sensitivity C-reactive protein; IL-6 = interleukin-6; suPAR = soluble urokinase plasminogen activator receptor

After adjustment for traditional risk factors, IL-6 and suPAR, but not hsCRP, remained independently associated with all-cause mortality in models further adjusted for E/e′ or GLS (Fig. 1). These associations were consistent when MACE was analyzed as the outcome (Supplemental Fig. 2).

**Fig. 2:**
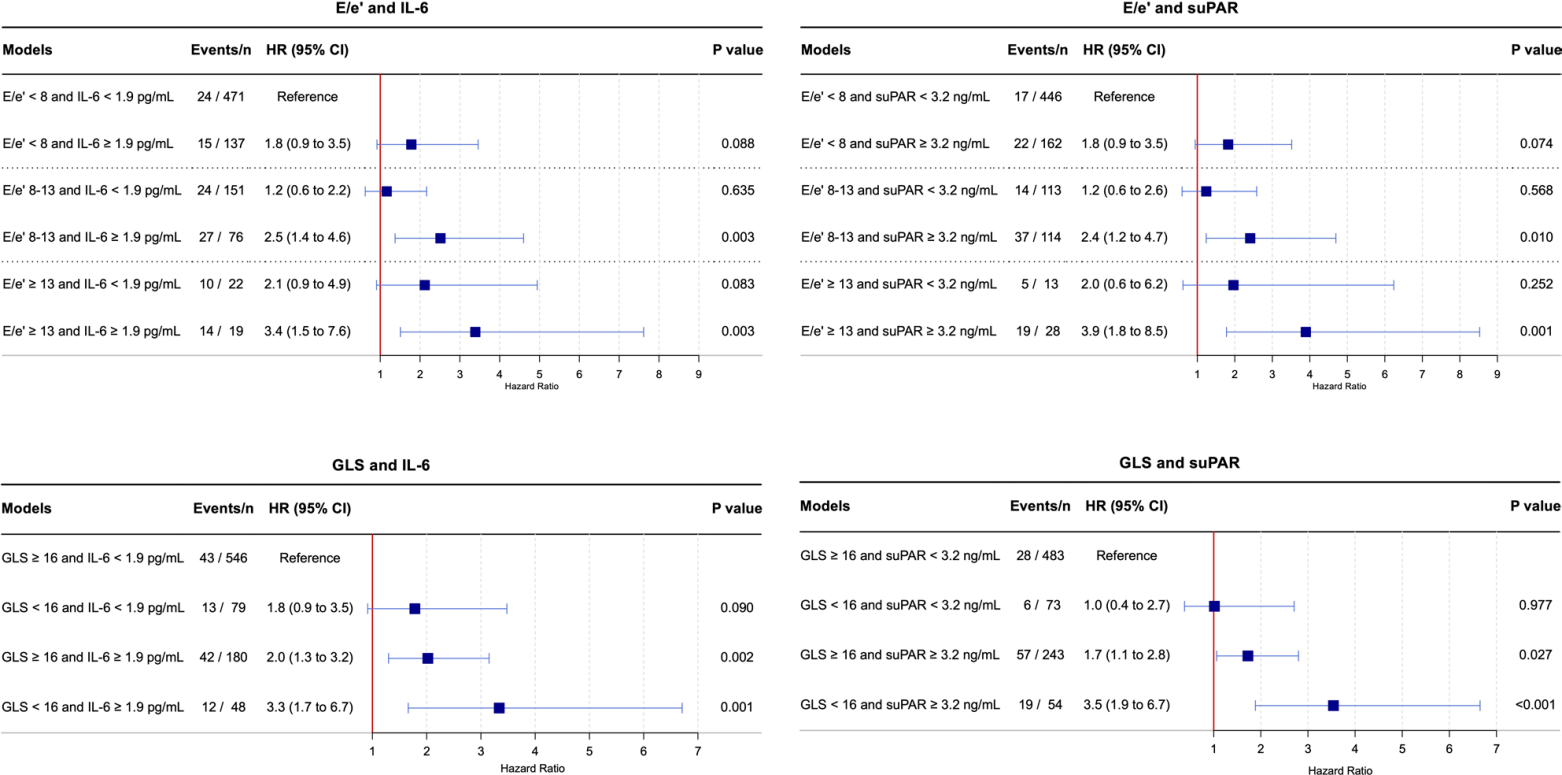
Combined effect of echocardiographic parameters and inflammatory biomarkers on all-cause mortality. HRs are adjusted for Steno T1 Risk Engine variables: age, sex, systolic blood pressure, duration of diabetes, HbA1c, low-density lipoprotein, estimated glomerular filtration rate, albuminuria status, smoking, and physical activity. CI = confidence interval; E/e’ = ratio of early mitral inflow velocity to early diastolic mitral annular velocity; GLS = global longitudinal strain; HR = hazard ratio; hsCRP = high-sensitivity C-reactive protein; IL-6 = interleukin-6; suPAR = soluble urokinase plasminogen activator receptor

To explore potential additive prognostic value, E/e′ and GLS were subsequently combined with IL-6 and suPAR, whereas hsCRP was not included, given its lack of independent association with mortality and MACE. Figure 2 shows that the highest risk of all-cause mortality was observed when elevations in E/e′ or impaired GLS coexisted with elevated IL-6 or suPAR. No association between E/e′ or GLS and all-cause mortality was observed among individuals without elevated IL-6 or suPAR. Corresponding findings were seen with MACE as the outcome. (Supplemental Fig. 3).

### Incremental prognostic value of E/e’ and inflammatory biomarkers

The addition of E/e’, GLS, or an inflammatory biomarker individually improved the performance of the Steno Type 1 Risk Engine (Table 2). Combining an echocardiographic parameter with an inflammatory biomarker resulted in a more pronounced improvement in C-statistic, indicating a synergistic effect, although the NRI gains were more modest in some models. Results were similar for MACE, particularly for models including E/e’ (Supplemental Table 2).

**Table 2:** Incremental prognostic value of combining E/e’ or GLS with IL-6 or SuPAR for all-cause mortality prediction

	C-statistics (95% CI)	NRI (95% CI)
ST1RE	0.839 (0.806 to 0.872)	Reference
ST1RE + E/e’ ≥ 8	0.843 (0.811 to 0.875)	23% (4% to 43%)
ST1RE + GLS < 16%	0.843 (0.810 to 0.876)	29% (9% to 48%)
ST1RE + suPAR ≥ 3.2 ug/mL	0.844 (0.811 to 0.877)	62% (42% to 81%)
ST1RE + IL-6 ≥ 1.9 pg/mL	0.844 (0.813 to 0.875)	52% (33% to 72%)
ST1RE + E/e’ ≥ 8 + suPAR ≥ 3.2 ug/mL	0.868 (0.832 to 0.904)	19% (-4% to 42%)
ST1RE + E/e’ ≥ 8 + IL-6 ≥ 1.9 pg/mL	0.887 (0.856 to 0.918)	86% (61% to 111%)
ST1RE + GLS < 16% + suPAR ≥ 3.2 ug/mL	0.869 (0.821 to 0.917)	63% (33% to 92%)
ST1RE + GLS < 16% + IL-6 ≥ 1.9 pg/mL	0.866 (0.828 to 0.904)	29% (6% to 52%)

The Steno T1 Risk Engine includes age, sex, systolic blood pressure, duration of diabetes, HbA1c, low-density lipoprotein, estimated glomerular filtration rate, albuminuria, smoking, and physical activity

C, confidence interval; GLS, global longitudinal strain; IL-6, interleukin-6; NRI, net reclassification improvement; ST1RE, Steno T1 Risk Engine; suPAR, soluble urokinase plasminogen activator receptor

### Sensitivity analyses

Sensitivity analyses yielded consistent associations with all-cause mortality (Supplemental Fig. 4) and MACE (Supplemental Fig. 5) when applying alternative thresholds for IL-6 (≥ 5.2 pg/mL) and suPAR (≥ 4.0 ng/mL). Improvements in both NRI and C-statistics were preserved under these alternate thresholds, with larger gains observed for the combined models (Supplemental Tables 3 and 4). Findings remained robust when adjusting for statins use, angiotensin-converting enzyme inhibitor therapy, and angiotensin II receptor blocker use across all models. Restricted cubic spline analyses demonstrated graded associations for E/e’, GLS, and inflammatory biomarkers, supporting the appropriateness of the categorical thresholds used in the primary analyses (Supplemental Fig. 6). No significant sex-specific differences in associations with all-cause mortality or MACE were observed.

## Discussion

In this prospective cohort study, we evaluated the long-term prognostic value of combining echocardiographic parameters of subclinical myocardial dysfunction (E/e’ or GLS) with systemic inflammatory biomarkers (IL-6, suPAR, or hsCRP) for the prediction of all-cause mortality and MACE in 876 individuals with type 1 diabetes, LVEF ≥ 50%, and no known cardiovascular or inflammatory disease. Our main findings were: First, elevated IL-6 and suPAR, but not hsCRP, were associated with all-cause mortality and MACE independent of traditional risk factors, E/e’, and GLS. Second, the risk of death and MACE was markedly higher when elevations in IL-6 or suPAR coexisted with elevated E/e’ or impaired GLS. Third, the combined inclusion of echocardiographic parameters and inflammatory biomarkers resulted in an improvement in risk discrimination of the Steno T1 Risk Engine beyond the incremental value of E/e′, GLS, IL-6, or suPAR individually.

### Echocardiographic risk stratification in type 1 diabetes

The findings suggest that integrating echocardiographic assessment with inflammatory biomarkers may enhance the identification of high-risk individuals with type 1 diabetes. In studies with shorter follow-up, we have previously demonstrated that echocardiographic parameters improve prediction in type 1 diabetes. These include E/e’ [13], global longitudinal strain [13], myocardial performance index [27], and E/e’ strain rate [28]. In the present study, GLS remained associated with all-cause mortality over long-term follow-up and independent of inflammatory biomarkers. Nevertheless, E/e’ demonstrated superior prognostic value overall. Indeed, diastolic abnormalities often occur early, and E/e’ has the advantages of being more accessible, easier to acquire, and widely clinically implemented. It has remained a cornerstone of assessing left ventricular diastolic dysfunction and heart failure with preserved ejection fraction (HFpEF) in international guidelines since 2005 [8, 29–33].

Despite their established clinical relevance, the implementation of E/e’ or GLS assessment in routine clinical practice in individuals with diabetes remains limited. This underutilization likely reflects several barriers: First, most clinicians are unaware of the prognostic value of echocardiography in diabetes. Second, logistical constraints related to access to echocardiography and scheduling may hinder its widespread use. Third, the cost of echocardiography and the need for trained personnel can be prohibitive. A targeted approach, directing echocardiographic evaluations to high-risk individuals, may mitigate these barriers by optimizing resource utilization. This targeted approach ensures the benefits of echocardiography outweigh the challenges associated with its implementation. Consistent with this approach, we found that E/e’ and GLS were rarely associated with adverse outcomes in the absence of elevated inflammatory biomarkers. Thus, inflammatory biomarkers may help guide selective echocardiographic screening in individuals with type 1 diabetes, thereby enhancing clinical efficiency and precision.

### Chronic inflammation as a link between diabetes and cardiovascular disease

Type 1 diabetes is characterized by chronic low-grade inflammation, as reflected by elevated circulating levels of IL-6, suPAR, and hsCRP compared with healthy individuals [34–36]. Chronic inflammation also plays a central role in the pathogenesis of heart failure (particularly HFpEF) and atherosclerosis, which together account for a substantial proportion of diabetes-related mortality. In HFpEF, low-grade systemic chronic inflammation is associated with adverse myocardial remodeling, leading to diastolic dysfunction and ultimately clinical heart failure [37]. Elevated inflammatory biomarkers are often observed in these patients, indicating a systemic inflammatory state that exacerbates cardiac stiffness and impairs relaxation [15]. In parallel, inflammatory pathways contribute to atherosclerosis through endothelial activation, immune cell infiltration, and plaque instability [38–40].

Chronic inflammation, therefore, represents a shared biological mechanism linking diabetes to cardiovascular disease. Indeed, we have previously shown that inflammatory biomarkers, particularly when combined, improve risk prediction of all-cause mortality and MACE in type 1 diabetes [20]. More recently, large studies have shown that multi-biomarker strategies outperform single-biomarker models for cardiovascular risk stratification [17, 41]. Consistent with these observations, our findings demonstrate that combining E/e′ or GLS with either IL-6 or suPAR improved the prognostic performance of the Steno Type 1 Risk Engine. This likely reflects the frequent but clinically silent presence of subclinical cardiovascular disease in individuals with type 1 diabetes.

Our findings align with current European Society of Cardiology guidelines, which recommend cardiovascular risk assessment in all patients with diabetes [6]. Importantly, we confirm these recommendations and extend beyond that. We demonstrate that even among those without known cardiovascular disease and LVEF ≥ 50%, assessment of subclinical myocardial dysfunction and inflammation, both individually and particularly in combination, can identify high-risk individuals. Notably, this applies not only to those with clearly abnormal E/e′ values (≥ 13), but also to those with intermediate values (8–13), which may be considered mildly elevated depending on age.

### Clinical implications

Early identification of individuals at high risk of adverse outcomes offers clinical potential to improve long-term prognosis in type 1 diabetes. Our findings suggest that combining a single echocardiographic parameter (E/e’ or GLS) with a biomarker of chronic inflammation (IL-6 or suPAR) may enable clinicians to distinguish between high and low risks of all-cause mortality and MACE. This approach could support targeted intensification of preventive strategies in high-risk individuals while providing reassurance to those at low risk.

Importantly, the high-risk phenotypes identified in this study may also be relevant in the context of emerging anti-inflammatory therapies for major diabetes-related complications. Ongoing phase III trials are evaluating IL-6 inhibition in atherosclerotic cardiovascular disease (NCT05021835) and heart failure (NCT05636176), while anti-suPAR therapies are under investigation in kidney disease (NCT06466135). All three conditions are major causes of morbidity in type 1 diabetes. In contrast, hsCRP is not causally associated with atherosclerosis and is currently not a direct therapeutic target [42]. In addition, the ongoing FINE-ONE trial (NCT05901831) is evaluating finerenone, a non-steroidal mineralocorticoid receptor antagonist with anti-inflammatory properties, for cardiorenal protection in individuals with type 1 diabetes [43]. Recent landmark trials have further demonstrated that inhibiting chronic inflammation is beneficial in type 1 diabetes and reduces cardiovascular risk in high-risk populations [16, 39, 40, 44, 45]. The proactive approach to identifying high-risk individuals presented in this study may not only improve individual patient care but hopefully also support broader efforts to reduce the premature mortality burden associated with type 1 diabetes.

### Strengths and limitations

This study’s key strengths include its large sample size, balanced sex distribution, planned and 100% complete long-term (~ 15 years) follow-up, and standardized in-person clinical assessment for all participants. The inclusion of individuals with type 1 diabetes without known cardiovascular disease, LVEF ≥ 50%, and no use of anti-inflammatory medication minimizes confounding from overt cardiovascular or inflammatory conditions and allows a focused evaluation of subclinical myocardial dysfunction and systemic inflammation. These design features enhance internal validity and support generalizability to similar populations without established cardiovascular or inflammatory disease, which represent the majority of individuals with type 1 diabetes. The use of established echocardiographic measures and multiple inflammatory biomarkers further strengthens the robustness and clinical relevance of the findings.

Several limitations should be acknowledged. First, the observational design precludes causal inference. Second, 67% of IL-6 values were below the assay detection limit and required imputation; however, model assumptions were met, and sensitivity analyses using alternative IL-6 thresholds (including ≥ 5.2 pg/mL) yielded consistent results. Importantly, this alternative cut-off would have been identical regardless of the imputation approach, supporting the robustness of the findings. Third, residual confounding cannot be excluded. Fourth, as we did not develop or recalibrate a new prediction model, cross-validation was not performed; consequently, improvements in NRI and C-statistics may be overestimated and should be interpreted cautiously. Fifth, limited numbers in some strata reduce precision and warrant cautious interpretation. Sixth, cause-specific mortality was not assessed due to lower completeness and quality of registry data compared with all-cause mortality, and the relatively small number of cause-specific events (e.g., cardiovascular or cancer deaths) would have limited statistical power. Therefore, all-cause mortality was selected as the most reliable endpoint. Seventh, the single-center design, predominantly Danish population from the Capital Region, and absence of gender identity data may limit generalizability to other ethnic, geographic, and gender-diverse populations. Finally, the lack of repeated biomarker and echocardiographic measurements precluded assessment of temporal changes in risk markers.

## Conclusions

The combined assessment of subclinical left ventricular dysfunction by echocardiography and chronic inflammation synergistically identifies individuals with type 1 diabetes (without known cardiovascular disease, LVEF ≥ 50%, and not receiving anti-inflammatory medication) who are at high risk of all-cause mortality and MACE over 14.5 years of follow-up. Elevated E/e’ or impaired GLS in conjunction with increased IL-6 or suPAR levels provides prognostic information beyond traditional risk factors and improves risk discrimination.

This study underscores the distinct yet complementary contributions of subclinical myocardial dysfunction and chronic inflammation to adverse outcomes in type 1 diabetes and supports an integrated, biology-informed approach to personalized risk stratification and therapeutic targeting. Further studies are warranted to validate these observations and to determine whether targeted interventions in individuals with concurrent subclinical left ventricular dysfunction and elevated inflammatory biomarkers can mitigate long-term risk.

### Translational outlook

This study proposes a pragmatic approach for identifying high-risk individuals with type 1 diabetes by integrating inflammatory biomarker profiling with echocardiographic assessment of subclinical left ventricular dysfunction. As routine echocardiography is neither feasible nor necessary for all patients, inflammatory biomarkers may function as an initial prescreening tool to identify those most likely to benefit most from targeted echocardiographic evaluation. This targeted approach could optimize resource utilization while enabling earlier and more precise risk stratification. Furthermore, as anti-inflammatory therapies for major diabetes-related complications advance into phase II and III trials, these findings may also help inform patient selection and contribute to the development of biomarker-guided preventive strategies.

Ethics declarations.

## Supplementary Information

Below is the link to the electronic supplementary material.

Supplementary file 1

## Data Availability

The data underlying this article cannot be shared publicly for the privacy of individuals who participated in the study. The data will be shared at a reasonable request to the corresponding author.

## Abbreviations

GLS: Global longitudinal strain
HFpEF: Heart failure with preserved ejection fraction
hsCRP: High-sensitivity C-reactive protein
IL-6: Interleukin-6
IQR: Interquartile range
LVEF: Left ventricular ejection fraction
MACE: Major adverse cardiovascular events
NRI: Net reclassification improvement
suPAR: Soluble urokinase plasminogen activator receptor
