# Supplementary material for: Echocardiography and inflammatory biomarkers for predicting mortality and major adverse cardiovascular events in type 1 diabetes
Source: Cardiovasc Diabetol. 2026 Feb 3;25:35. doi: 10.1186/s12933-025-03071-2 (PMC12879427; doi:10.1186/s12933-025-03071-2)
Supplement: Supplementary file 1 [file 12933_2025_3071_MOESM1_ESM.docx]

| **Supplemental Table 2.** Incremental prognostic value of combining E/e’ or GLS with IL-6 or suPAR for MACE prediction | | |
| --- | --- | --- |
|  | **C-statistics (95% CI)** | **NRI (95% CI)** |
| ST1RE | 0.781 (0.749 to 0.813) | Reference |
| ST1RE + E/e’ ≥8 | 0.789 (0.759 to 0.819) | 23% (6% to 39%) |
| ST1RE + GLS <16% | 0.784 (0.753 to 0.815) | 14% (-3% to 31%) |
| ST1RE + suPAR ≥3.2 ug/mL | 0.790 (0.758 to 0.822) | 55% (39% to 72%) |
| ST1RE + IL-6 ≥1.9 pg/mL | 0.785 (0.754 to 0.816) | 42% (26% to 58%) |
| ST1RE + E/e’ ≥8 + suPAR ≥3.2 ug/mL | 0.843 (0.809 to 0.877) | 69% (49% to 89%) |
| ST1RE + E/e’ ≥8 + IL-6 ≥1.9 pg/mL | 0.835 (0.799 to 0.871) | 86% (65% to 107%) |
| ST1RE + GLS <16% + suPAR ≥3.2 ug/mL | 0.817 (0.770 to 0.864) | 55% (30% to 78%) |
| ST1RE + GLS <16% + IL-6 ≥1.9 pg/mL | 0.787 (0.746 to 0.828) | 25% (7% to 43%) |
| The Steno T1 Risk Engine includes age, sex, systolic blood pressure, duration of diabetes, HbA1c, low-density lipoprotein, estimated glomerular filtration rate, albuminuria, smoking, and physical activity.  CI = confidence interval; GLS = global longitudinal strain; IL-6 = interleukin-6; MACE = major adverse cardiovascular events; NRI = net reclassification improvement; ST1RE = Steno T1 Risk Engine;  suPAR = soluble urokinase plasminogen activator receptor. | | |

| Supplemental Table 1. Baseline characteristics by E/e′ combined with hsCRP | | | | | |
| --- | --- | --- | --- | --- | --- |
|  | | **All** | **E/e’ <8  & hsCRP <2.0** | **E/e’ ≥8  & hsCRP ≥2.0** | **P value** |
|  | | (n=876) | (n=375) | (n=144) |  |
| Age, years, median (IQR) | | 50 [39-60] | 46 [37-56] | 59 [52-67] | <0.001 |
| Male sex, n (%) | | 447 (51) | 229 (61) | 58 (40) | <0.001 |
| Body mass index, kg/m^2^, median (IQR) | | 25 [23-28] | 24 [22-26] | 27 [25-30] | <0.001 |
| Diabetes duration, years, median (IQR) | | 25 [15-36] | 21 [12-33] | 36 [26-45] | <0.001 |
| Systolic blood pressure, mmHg, median (IQR) | | 132 [122-142] | 128 [120-140] | 140 [130-154] | <0.001 |
| eGFR, mL/min/1.73m^2^, median (IQR) | | 89 [ 76-102] | 93 [ 80-107] | 79 [62-94] | <0.001 |
| Hemoglobin A1c, mmol/L, median (IQR) | | 64 [57-74] | 63 [55-70] | 66 [58-73] | 0.033 |
| Hemoglobin A1c, %, median (IQR) | | 8.0 [7.4-8.9] | 7.9 [7.2-8.6] | 8.2 [7.5-8.8] | 0.033 |
| Total cholesterol, mmol/L, median (IQR) | | 4.8 [4.2-5.3] | 4.7 [4.1-5.2] | 4.8 [4.3-5.3] | 0.053 |
| Low-density lipoprotein, mmol/L, median (IQR) | | 2.5 [2.1-3.0] | 2.5 [2-3] | 2.4 [2.0-2.9] | 0.264 |
| Interleukin-6, pg/mL, median (IQR) | | 1.0 [1.0-2.1] | 1.0 [1.0-1.0] | 1.2 [1.0-5.5] | <0.001 |
| suPAR, ng/mL, median (IQR) | | 2.8 [2.3-3.5] | 2.7 [2.2-3.2] | 3.4 [2.7-4.6] | <0.001 |
| hsCRP, mg/L, median (IQR) | | 1.7 [0.7-3.6] | 0.7 [0.4-1.2] | 4.1 [2.8-6.4] | <0.001 |
| Current smoking, n (%) | | 485 (55) | 194 (52) | 96 (67) | 0.009 |
| Albuminuria, n (%) | | |  |  | <0.001 |
|  | Mildly increased | 631 (72) | 308 (82) | 74 (51) |  |
|  | Moderately increased | 76 (9) | 14 (4) | 30 (21) |  |
|  | Severely increased | 169 (19) | 53 (14) | 40 (28) |  |
| Medications at inclusion, n (%) | | |  |  |  |
|  | Statins | 365 (42) | 114 (30) | 94 (65) | <0.001 |
|  | ACE-I/ARB | 387 (44) | 125 (33) | 102 (71) | <0.001 |
|  | Beta-blockers | 34 (4) | 41 (1) | 21 (15) | <0.001 |
|  | Diuretics | 217 (25) | 47 (13) | 80 (56) | <0.001 |
| Echocardiography | |  |  |  |  |
|  | LVEF, %, median (IQR) | 58 [55-61] | 58 [55-61] | 59 [55-62] | 0.080 |
|  | GLS, %, median (IQR) | 18 [17-20] | 19 [17-20] | 18 [16-19] | <0.001 |
|  | E/e', median (IQR) | 7 [5-8] | 6 [5-7] | 10 [9-12] | <0.001 |
|  | e’ lateral, cm/s, median (IQR) | 13 [10-16] | 14 [12-17] | 9 [8-10] | <0.001 |
|  | Left atrial volume, mL/m² | 29 [26-34] | 30 [26-34] | 29 [26-34] | 0.256 |
| P value for comparison between low-low and high-high groups.  ACE-I = angiotensin-converting enzyme inhibitor; ARB = angiotensin receptor blocker; hsCRP = High-sensitivity C-reactive protein; eGFR = estimated glomerular filtration rate; GLS = global longitudinal strain; IL-6 = interleukin-6; IQR = interquartile range; LVEF = left ventricular ejection fraction; suPAR = soluble urokinase plasminogen activator receptor. | | | | | |

| **Supplemental Table 2.** Incremental prognostic value of combining E/e’ or GLS with IL-6 or suPAR for MACE prediction | | |
| --- | --- | --- |
|  | **C-statistics (95% CI)** | **NRI (95% CI)** |
| ST1RE | 0.781 (0.749 to 0.813) | Reference |
| ST1RE + E/e’ ≥8 | 0.789 (0.759 to 0.819) | 23% (6% to 39%) |
| ST1RE + GLS <16% | 0.784 (0.753 to 0.815) | 14% (-3% to 31%) |
| ST1RE + suPAR ≥3.2 ug/mL | 0.790 (0.758 to 0.822) | 55% (39% to 72%) |
| ST1RE + IL-6 ≥1.9 pg/mL | 0.785 (0.754 to 0.816) | 42% (26% to 58%) |
| ST1RE + E/e’ ≥8 + suPAR ≥3.2 ug/mL | 0.843 (0.809 to 0.877) | 69% (49% to 89%) |
| ST1RE + E/e’ ≥8 + IL-6 ≥1.9 pg/mL | 0.835 (0.799 to 0.871) | 86% (65% to 107%) |
| ST1RE + GLS <16% + suPAR ≥3.2 ug/mL | 0.817 (0.770 to 0.864) | 55% (30% to 78%) |
| ST1RE + GLS <16% + IL-6 ≥1.9 pg/mL | 0.787 (0.746 to 0.828) | 25% (7% to 43%) |
| The Steno T1 Risk Engine includes age, sex, systolic blood pressure, duration of diabetes, HbA1c, low-density lipoprotein, estimated glomerular filtration rate, albuminuria, smoking, and physical activity.  CI = confidence interval; GLS = global longitudinal strain; IL-6 = interleukin-6; MACE = major adverse cardiovascular events; NRI = net reclassification improvement; ST1RE = Steno T1 Risk Engine;  suPAR = soluble urokinase plasminogen activator receptor. | | |

| **Supplemental Table 3.** Incremental prognostic value of combining E/e’ or GLS with IL-6 or suPAR for all-cause mortality prediction (alternative thresholds) | | |
| --- | --- | --- |
|  | **C-statistics (95% CI)** | **NRI (95% CI)** |
| ST1RE | 0.839 (0.806 to 0.872) | Reference |
| ST1RE + E/e’ ≥8 | 0.843 (0.811 to 0.875) | 23% (4% to 43%) |
| ST1RE + GLS <16 | 0.843 (0.810 to 0.876) | 29% (9% to 48%) |
| ST1RE + suPAR ≥4.0 ug/mL | 0.841 (0.808 to 0.874) | -8% (-28% to 12%) |
| ST1RE + IL-6 ≥5.2 pg/mL | 0.841 (0.808 to 0.874) | 30% (12% to 49%) |
| ST1RE + E/e’ ≥8 + suPAR ≥4.0 ug/mL | 0.877 (0.840 to 0.914) | 38% (13% to 64%) |
| ST1RE + E/e’ ≥8 + IL-6 ≥5.2 pg/mL | 0.865 (0.824 to 0.906) | 37% (9% to 64%) |
| ST1RE + GLS <16 + suPAR ≥4.0 ug/mL | 0.855 (0.815 to 0.895) | 62% (36% to 87%) |
| ST1RE + GLS <16 + IL-6 ≥5.2 pg/mL | 0.856 (0.816 to 0.896) | 34% (12% to 56%) |
| The Steno T1 Risk Engine includes age, sex, systolic blood pressure, duration of diabetes, HbA1c, low-density lipoprotein, estimated glomerular filtration rate, albuminuria, smoking, and physical activity.  CI = confidence interval; GLS = global longitudinal strain; IL-6 = interleukin-6; NRI = net reclassification improvement; ST1RE = Steno T1 Risk Engine; suPAR = soluble urokinase plasminogen activator receptor. | | |

| **Supplemental Table 4.** Incremental prognostic value of combining E/e’ with IL-6 or suPAR for MACE prediction (alternative thresholds) | | |
| --- | --- | --- |
|  | **C-statistics (95% CI)** | **NRI (95% CI)** |
| ST1RE | 0.781 (0.749 to 0.813) | Reference |
| ST1RE + E/e’ ≥8 | 0.789 (0.759 to 0.819) | 23% (6% to 39%) |
| ST1RE + GLS <16 | 0.784 (0.753 to 0.815) | 14% (-3% to 31%) |
| ST1RE + suPAR ≥4.0 ug/mL | 0.781 (0.749 to 0.813) | -16% (-33% to 0%) |
| ST1RE + IL-6 ≥5.2 pg/mL | 0.788 (0.757 to 0.819) | 20% (5% to 35%) |
| ST1RE + E/e’ ≥8 + suPAR ≥4.0 ug/mL | 0.836 (0.800 to 0.872) | 71% (50% to 92%) |
| ST1RE + E/e’ ≥8 + IL-6 ≥5.2 pg/mL | 0.828 (0.790 to 0.866) | 53% (30% to 75%) |
| ST1RE + GLS <16 + suPAR ≥4.0 ug/mL | 0.791 (0.751 to 0.831) | 44% (23% to 65%) |
| ST1RE + GLS <16 + IL-6 ≥5.2 pg/mL | 0.788 (0.748 to 0.828) | 16% (1% to 31%) |
| The Steno T1 Risk Engine includes age, sex, systolic blood pressure, duration of diabetes, HbA1c, low-density lipoprotein, estimated glomerular filtration rate, albuminuria, smoking, and physical activity.  CI = confidence interval; IL-6 = interleukin-6; MACE = major adverse cardiovascular events; NRI = net reclassification improvement; ST1RE = Steno T1 Risk Engine;  suPAR = soluble urokinase plasminogen activator receptor. | | |
